# The Potential Benefit of Monitoring Oxidative Stress and Inflammation in the Prevention of Non-Communicable Diseases (NCDs)

**DOI:** 10.3390/antiox10010015

**Published:** 2020-12-27

**Authors:** Neda Seyedsadjadi, Ross Grant

**Affiliations:** 1 Australasian Research Institute, Sydney Adventist Hospital, Sydney, NSW 2076, Australia; n.seyedsadjadi@unsw.edu.au; 2 School of Biotechnology and Biomolecular Sciences, Faculty of Science, University of New South Wales, Sydney, NSW 2052, Australia; 3 Department of Pharmacology, School of Medical Sciences, Faculty of Medicine, University of New South Wales, Sydney, NSW 2052, Australia; 4 Sydney Adventist Hospital Clinical School, University of Sydney, Sydney, NSW 2076, Australia

**Keywords:** oxidative stress, inflammation, biomarkers, non-communicable disease, lifestyle, prevention, clinical application

## Abstract

The significant increase in worldwide morbidity and mortality from non-communicable diseases (NCDs) indicates that the efficacy of existing strategies addressing this crisis may need improvement. Early identification of the metabolic irregularities associated with the disease process may be a key to developing early intervention strategies. Unhealthy lifestyle behaviours are well established drivers of the development of several NCDs, but the impact of such behaviours on health can vary considerably between individuals. How can it be determined if an individual’s unique set of lifestyle behaviours is producing disease? Accumulating evidence suggests that lifestyle-associated activation of oxidative and inflammatory processes is primary driver of the cell and tissue damage which underpins the development of NCDs. However, the benefit of monitoring subclinical inflammation and oxidative activity has not yet been established. After reviewing relevant studies in this context, we suggest that quantification of oxidative stress and inflammatory biomarkers during the disease-free prodromal stage of NCD development may have clinical relevance as a timely indicator of the presence of subclinical metabolic changes, in the individual, portending the development of disease. Monitoring markers of oxidative and inflammatory activity may therefore enable earlier and more efficient strategies to both prevent NCD development and/or monitor the effectiveness of treatment.

## 1. Introduction

Many benefits have arisen from recent advances in modern technology especially in the field of medical sciences, enhancing the effectiveness of numerous disease treatments with the consequent improvement in both quality of life and extension in lifespan. However, living in modern societies has proven to be a double-edged sword, with several elements of the popular Western lifestyle actually contributing to the development of age-related chronic diseases, in particular the non-communicable diseases (NCDs) such as cardiovascular diseases, diabetes, neurodegenerative dementia and cancer [1,2,3]. While the lifespan of an individual suffering from any of these diseases may be extended through the application of an efficient disease management strategy [4], the cumulative effects of unaddressed poor lifestyle choices will promote a state of chronic morbidity and make them more vulnerable to ongoing development of degenerative changes and further NCDs [5,6].

NCDs are the leading causes of morbidity and mortality worldwide. Of the global reported deaths of 56.4 million in 2015, 70%, or 39.5 million, were due to NCDs including 14 million people who died too young before the age of 70 [7]. The annual mortality from NCDs is projected to increase to 55 million throughout the world by the year 2030 [8]. This is a costly outcome in terms of both personal morbidity and financial outlay as it is estimated that NCDs alone will drain over USD 60 trillion from the global economy from 2011 to 2030 [9].

The prevention of NCDs is a global challenge assigned a high priority by the World Health Organisation (WHO, Geneva, Switzerland) [8]. NCDs generally have a long prodromal stage often taking many years to develop. As a result, the potential for developing an NCD increases as the population ages. This rise in disease morbidity linked to the ageing population poses important challenges to health care systems globally [10]. Therefore, an effective early preventive strategy is an ideal approach to slow or even halt lifestyle-linked degenerative processes. Application of such a strategy in a healthcare model that places emphasis on disease prevention, rather than just disease management, appears necessary if the burgeoning NCD problem is to be averted [8]. The benefits of implementing a successful preventive health strategy are immense as it has been estimated that around 80% of type 2 diabetes, 80% of premature heart disease and stroke, and 40% of cancers are preventable [11]. While the importance of prevention has been highlighted throughout the literature for many years, integration of a successful preventive strategy for NCDs in modern health care systems is still not apparent. Although successful in controlling many of the world’s infectious diseases, the WHO is still searching for an effective way to control runaway NCDs [8].

As mentioned, NCDs are diseases of generally slow progression with clinical symptoms only becoming apparent after considerable cellular damage has occurred in target tissues. Research now supports the argument that this damage is largely influenced by biochemical changes associated with a redox imbalance producing a state of “chronic oxidative stress” often accompanied or linked to inflammation [12,13,14]. Oxidative stress has been identified as a critical step in the initiation and the development of the pathophysiological changes associated with several NCDs [15,16]. Elevated intracellular/plasma levels of free radicals (molecules with an unpaired valence electron), such as reactive oxygen species (ROS), can cause severe damage to cellular structures including lipids, proteins and DNA molecules, disrupting their functions and leading to fundamental damage to the entire cell [16,17]. Chronic exposure to these oxidative processes creates a subclinical state of progressive cell damage which accumulates over time. This damage facilitates tissue remodelling (e.g., to vascular endothelium in CVD) and eventually results in selective system failures and the dysfunctional state known as disease [14,16,18,19]. Therefore, measurement of biochemical markers associated with oxidative stress and inflammation may be useful in identifying an individual’s early biochemical shift toward disease, especially in the initial prodromal stage. The possibility of early (preclinical) detection, afforded by use of oxidative stress/inflammatory biomarkers, may also enable a more individualised and sensitive profiling compared to conventional population-based risk assessment tools that currently rely on downstream biomarkers [20,21].

In this paper, we review the available literature evidence on the importance of lifestyle-induced oxidative stress and inflammation in the development of NCDs and suggest that early correction of redox imbalances may be useful in arresting NCD development.

## 2. Lifestyle Links to NCDs

Unhealthy lifestyle behaviours are widely accepted as primary initiators of the disease processes through which NCDs develop [1,3,22]. Even though genetic factors have been shown to play a role, their contribution to NCDs has been estimated to be less than 10% [23]. Therefore, modifiable lifestyle behaviours are especially relevant as they are more amenable to intervention [11,24,25]. Epidemiological studies clearly identify lifestyle as a primary risk factor for the development of NCDs [1,11,26,27]. In one large cohort study, after a 20 year follow up, it was shown that unhealthy lifestyle behaviours including physical inactivity, alcohol intake, cigarette smoking and low intake of fruits and vegetables were associated with increased mortality risk of cardiovascular disease (CVD) and cancer [1]. Furthermore, in the European Prospective Investigation into Cancer and Nutrition, after a 7-year follow up, it was shown that practicing healthy lifestyle behaviours including performing physical activity, adhering to healthy dietary principles, not smoking, and having a body mass index (BMI) lower than 30, for 7 years was associated with 78% lower risk of developing a chronic disease (93% lower risk of diabetes, 81% lower risk of myocardial infarction, 50% lower risk of stroke, and 36% lower risk of cancer) [11]. Lifestyle behaviours are strongly linked to the development of NCDs, and as also suggested by others [28,29,30] a better understanding of the possible biochemical mechanisms through which these behaviours influence the disease progression will be helpful in informing effective preventive strategies.

## 3. Risk Scores and Lifestyle Behaviours

It has become increasingly apparent however that the interaction between lifestyle and the body’s biochemistry is multifaceted requiring complex modelling [22,31,32,33]. Unhealthy lifestyle behaviours are well known to cause metabolic changes in plasma levels of lipids and fasting glucose [33,34]. This has led many to develop various population-based risk scores composed of these biomarkers to identify groups at risk of NCDs and also to monitor the efficacy of any preventive approach [20,35,36,37,38,39]. For example, a number of risk scores are available for the prediction of coronary heart disease such as the Framingham risk score (FRS) [20,21], World Health Organization/International Society of Hypertension (WHO/ISH) risk prediction charts [38], American College of Cardiology/American Heart Association (ACC/AHA) pooled cohort equations [36], the third Joint British Societies’ risk calculator (RiskJBS) [37], and the Systematic Coronary Risk Evaluation (SCORE) [39]. While these are validated and widely used tools for the prediction of coronary heart disease, studies suggest that they tend to underestimate an individual’s actual risk, thereby potentially delaying initiation of an appropriate intervention such as lifestyle modification [35,40,41,42,43,44,45,46,47,48].

In regard to the assessment of total cardiovascular (CVD) risk at the population level, it has been shown that WHO/ISH charts underestimate the risk of Asian populations with high total CVD risk [44]. Furthermore, the value of FRS in identifying disease risk has been reported to be less clear in patients with concomitant inflammatory diseases, such as systemic lupus erythematosus (SLE) [45] and psoriatic arthritis [46], patients with diabetes [35], breast cancer [47] and metabolic syndrome [48]. While these diseases are associated with increased risk for premature cardiovascular disease, persons with these co-morbidities are estimated to have a low risk according to the FRS calculations. In addition, based on SCORE charts, younger individuals will always be categorised in the low risk category, even when all constituent risk factor levels are very unfavourable, and no combination of risk factors will place a 40-year old individual in the high risk category [39]. Thus, other risk biomarkers not currently included in these risk score calculations appear necessary to more accurately predict actual individual risk. Current risk score calculations include numerous risk factors such as age, gender, markers of dyslipidaemia, blood pressure, body-mass index (BMI), and history of diabetes and smoking [20,35,36,37,38,39]. However, these conventional risk factors cannot account for all cases of CVD [35,40,41,42,43,44,45,46,47,48]. This is, at least partially, due to the fact that the traditional biomarkers such as total cholesterol (TC) and low density lipoprotein cholesterol (LDL-C) are not primary initiators of the disease process, but rather mediators of downstream pathology [49]. This suggests that other, currently less conventional biomarkers, that are more upstream of the primary damage process, need to be included in the risk score calculations in order to improve the risk estimation potency of a probable NCD at the individual level.

## 4. Oxidative Stress Is Upstream of Conventional NCD Biomarkers

**Figure 1:**
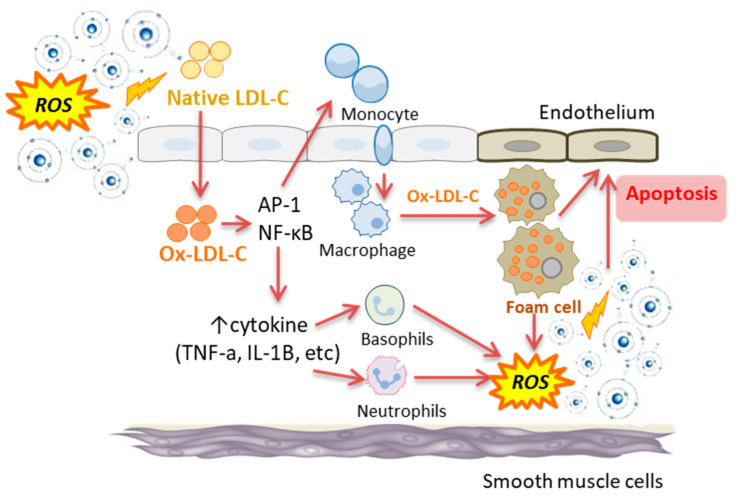
The interplay between oxidative stress and inflammatory processes (i.e., NFκB and cytokine activity) in the development of endothelial cell damage and apoptosis. ROS: reactive oxygen species; Ox-LDL-C: oxidised low density lipoprotein cholesterol; AP-1: activator protein-1; NF-κB: nuclear factor-kappa B; TNF-α: tumour necrosis factor- α; IL-1β: interleukin-1β.

There has been a growing appreciation in the literature of how non-conventional metabolic, oxidative stress and inflammatory biomarkers are more direct indicators of NCD pathologies than conventional biomarkers alone. For example, progression to type 1 diabetes has been associated with the downregulation of amino acid metabolites, sugar derivatives and fatty acids [29]. Several studies have also identified the critical role of oxidative and inflammatory processes in all phases of coronary artery disease, from foam cell formation to plaque rupture and thrombosis [50,51,52,53]. The concept originated from an in vitro observation that showed oxidised LDL-C but not native LDL-C, could increase the motility of monocytes via chemotactic activity [54]. The recruitment of monocytes/macrophages to the damage site through enhanced chemotactic activity is a critical step in the initiation and progression of the damage to the endothelial cells in the arteries. Oxidised LDL, taken up by the macrophage scavenger system, if present in excess will transform the macrophages into a non-functional foam cell which subsequently forms part of the maturing plaque in the developing atherosclerotic lesion [19]. It also triggers inflammatory processes which further exacerbate an oxidative environment [55] (Figure 1).

In this context, studies in otherwise healthy individuals have shown significant associations between oxidative stress biomarkers including F2-isoprostanes, malondialdehyde (MDA), 8- hydroxy deoxyguanosine (8-OHdG), oxidised disulphide form of cystine (CySS), and the ratio of glutathione (GSH) to its oxidised disulphide form (GSSG), and subclinical indicators of CVD development such as carotid artery intima-media thickness (CIMT) and flow-mediated vasodilation (FMD), even after controlling for the conventional CVD risk markers, such as LDL-C, and FRS [18,56].

In addition, independent and strong correlations have been reported between the lipid peroxidation biomarkers hydroxy-eicosatetraenoic acids (HETEs) and F2-isoprostanes and angiographic evidence of CVD, even after adjustment for FRS. The inclusion of these biomarkers into a multivariate prediction model that also included the FRS has been shown to significantly improve the prediction of coronary heart disease [57].

In regard to inflammatory biomarkers, the independent association between plasma levels of inflammatory biomarkers, C-reactive protein (CRP) and interleukin (IL)-6, and CVD in multivariable analyses including conventional CVD risk factors have previously been shown [58]. Importantly, it has been reported that plasma CRP levels can help to estimate the risk of initial cardiovascular events and even improve CVD risk classification in individuals with intermediate risk [59,60]. Therefore, the measurement of oxidative stress and inflammatory biomarkers as part of routine assessment appears to have clinical relevance by increasing the sensitivity of current risk scores and providing an early indication of the presence of a (subclinical) disease process.

Importantly, the part played by oxidative stress in disease development is consistent with the oxidative stress theory of ageing which posits that the accumulation of unrepaired oxidative damage at the cellular level is a primary player in the development of the aging phenotype [61]. In support of this concept, a number of recent studies from our laboratory [62,63] and others [64] have confirmed an increased level of oxidative damage biomarkers in aged tissues. Thus, the association between ageing and increased incidence of NCDs is at least partly explained by a subclinical increase in oxidative and inflammatory potential and changes in related biomarkers which underpin and/or exacerbate development of several NCDs [14]. However, what is the relationship between oxidative stress and inflammation?

## 5. Association between Oxidative Stress and Inflammation

Inflammation is the body’s nonspecific and immediate response by the innate immune system that is initiated in an acute phase response to environmental challenges such as pathogens and physical or chemical injuries including free radical attack [65,66]. Indeed, increased free radical generation and a state of oxidative stress and inflammation are closely interrelated. Oxidative stress can cause the damage to tissue that can trigger an inflammatory response, which in turn, can be a direct inducer of oxidative stress [53,66]. In this context, oxidative stress has been shown to cause the activation of redox-sensitive transcription factors, activator protein-1 (AP-1) and nuclear factor-kappa B (NF-κB) [67]. These factors can induce inflammatory processes and activate the innate immune system with the subsequent recruitment and the accumulation of inflammatory cells such as macrophages, leukocytes and neutrophils to the damage site [68,69]. These inflammatory cells can then produce metabolites of soluble mediators such as arachidonic acid, chemokines and pro-inflammatory cytokines such as interleukin (IL)-1β, IL-6 and tumour necrosis factor (TNF)-α [70,71]. Elevated levels of these pro-inflammatory cytokines can result in hepatic production and secretion of acute-phase proteins such as C-reactive protein (CRP) and also can further recruit inflammatory cells to the damage site [72,73]. Importantly, the generation of oxygen and/or nitrogen free radicals is a characteristic property of activated immune cells to eliminate pathogens or other stimuli. In other words, the production of free radicals is the primary damaging agents while the cytokines and acute phase proteins produced are essentially signalling molecules.

It is important to note that cytokines themselves do not cause direct cellular damage. Indeed, chronic inflammation exerts its cellular side effects largely through excessive generation of free radicals and depletion of antioxidants [14,66,74]. Hence, the accumulation of activated immune cells at the damage site accelerates the generation of free radicals and ROS which further promote the ongoing oxidative stress. Therefore, oxidative stress triggers an inflammatory response, which can in turn amplify oxidative stress [53,66] (Figure 1).

## 6. What Is Redox Imbalance and How Does It Relate to Oxidative Stress?

After the discovery of the oxidation of organic molecules by hydrogen peroxide in the presence of Fe^++^ by Fenton in 1894 [75], a series of investigations began in this area which later became known as oxidative stress. The generation of reactive oxidative species (ROS), the main players in oxidative reactions, is a normal consequence of all forms of aerobic life. In mammals, about 95% of inspired oxygen (O_2_) is successfully reduced to CO_2_ and H_2_O, without the formation of toxic radicals. However, the remaining 5%, proceeds through univalent reduction, producing reactive oxygen species which include superoxide (O^2−^) radicals, hydroxyl radical (OH^−^), hydrogen peroxide (H_2_O_2_) and the peroxynitrite (OONO^−^) radical [76]. The major sources of ROS production is mitochondrion [77], peroxisomal oxidase [78], xanthine oxidase [79], nicotinamide adenine dinucleotide phosphate (NADPH) oxidase [80] and cytochrome P450 enzymes [81]. The generation of ROS is also the major way the immune system uses to attack and kill pathogens and digest cell debris [53,66,82]. Inflammatory cells such as macrophages and neutrophils contain the NADPH oxidase system that, when activated, produces large amounts of superoxide and other damaging radical species that in the case of chronic inflammation can eventually lead to pervasive collateral cellular damage. Thus, free radical damage is the primary driver of cellular damage resulting from inflammatory activity [53,66,82,83].

Importantly, some free radical species also play a role as second messengers in controlling significant cellular functions such as the production of chemical mediators, cellular division and migration [67]. However, as free radicals with at least one unpaired electron in their atomic structure, they can be harmful at supra physiological concentrations [], and are counterbalanced by a complex antioxidant defence system comprising enzymatic (e.g., superoxide dismutase, glutathione peroxidise, glutathione reductase, and catalase) and non-enzymatic sources (e.g., dietary vitamins and phytochemicals, and glutathione) [13].

In all living cells a delicate balance exists between the generation, and the elimination of ROS by the endogenous antioxidant defence system. This is referred to commonly as the redox balance. Generally, overproduction of ROS or a defect in the ability of the endogenous antioxidant defence system to potentially detoxify these reactive substances or to repair the resulting damage, can lead to a redox imbalance [84]. Redox imbalance contributes significantly to the development of widespread cellular oxidation. This situation has been defined as oxidative stress (a state of redox imbalance), which as mentioned can eventually result in cell damage and death, with the consequent tissue alterations linked to the development of NCDs [14,16].

## 7. The Link between Lifestyle Behaviours, Oxidative Stress and Inflammation

**Figure 2:**
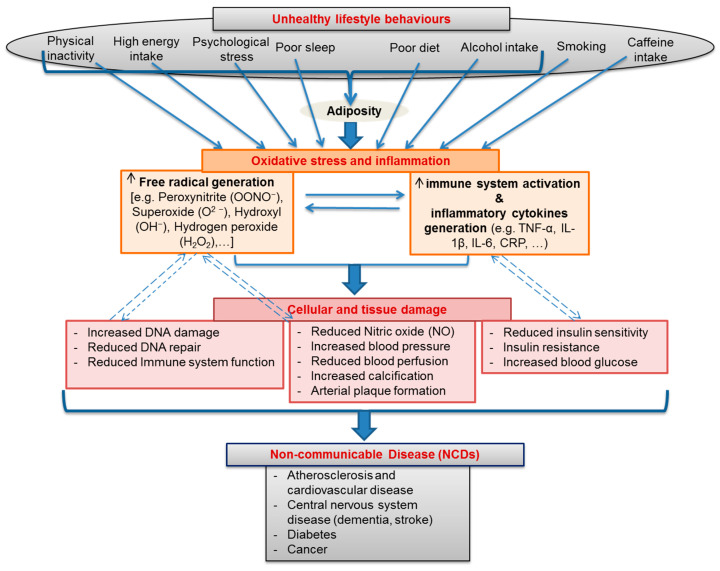
A schematic showing lifestyle behaviours as major drivers behind the development of an oxidative stressed and inflammatory state which in turn play primary roles in the chronic cellular and tissue damage that leads to the final development of multiple non-communicable diseases (NCDs).

As discussed above, a persuasive case is emerging for a pivotal role of oxidative damage and inflammation in the remodelling of tissue into its diseased phenotype at the biochemical and cellular levels [14,16,18,19]. Therefore, the assessment of oxidative and inflammatory biomarkers may improve disease stratification [57]. As previously mentioned, robust associations between the development of NCDs and unhealthy lifestyle behaviours have been known for some time [1,3,22]. Therefore, it is plausible that these two causal factors are interlinked. Indeed, there is a growing body of evidence showing that various lifestyle behaviours can induce a state of oxidative stress and inflammation which eventually leads to chronic NCDs [32,85,86] (Figure 2). In the following section, we provide evidence on how specific modifiable lifestyle behaviours have been linked to oxidative stress and inflammatory activity.

### 7.1. Adiposity and Total and Regional Body Fat

A positive energy balance, due to an imbalance between food (energy) intake and energy expenditure, can increase total body fat content, and visceral adipose tissue (VAT) fat mass, a key indicator of central obesity [87,88]. Total body fat and VAT have been reported to be positively associated with oxidative and inflammatory processes in several studies [89,90,91]. Indeed, ROS generation from NADPH oxidase activity has been detected in adipose tissue, and has been shown to progressively increase in aged adipose tissue [89,90]. There is also evidence that hypertrophic adipocytes can cause a state of local hypoxia due to their increased oxygen consumption [92]. Local hypoxia can increase the levels of total ROS and superoxide in particular, and decrease levels of key antioxidant enzymes, superoxide dismutase (SOD1 and SOD3) and catalase in adipocytes [90]. Furthermore, hypoxia has been shown to trigger the up-regulation of hypoxic-inducible factor (HIF) which has been linked to mitochondrial dysfunction [93] and activation of NF-κB with the subsequent production of inflammatory cytokines [94]. Another contributor to adipocyte-induced oxidative stress has been suggested to be alterations in gut microbiota that are accompanied by enhanced production of gut bacterial metabolite deoxycholic acid (DCA) [95]. DCA is known to damage DNA molecule through generation of ROS [96]. Visceral fat adipocytes can also significantly increase levels of plasma inflammatory biomarkers such as CRP [97], TNF-α [98] and IL-6 [99], either directly by releasing high amounts of these cytokines or indirectly by producing the inflammatory adipokines, leptin and resistin [91]. These inflammatory cytokines play a pivotal role in reducing insulin sensitivity and developing insulin resistant [100]. The persistent hyperglycaemia, secondary to insulin resistance, can further exacerbate the existing redox imbalance by inducing oxidative stress and contribute to beta cell destruction in type 2 diabetes [101].

### 7.2. Nutritional Behaviours

#### 7.2.1. Energy Intake

In addition to the mentioned indirect effects of high energy intake in promoting a state of oxidative stress and inflammation via increasing body weight and total body fat [89,90,91], energy intake can be directly involved in producing a state of oxidative stress and inflammation [102,103,104,105,106]. Boden et al., reported that feeding healthy men with a high calorie diet (6000 kcal/day) for one week can produce a rapid onset of systemic oxidative stress detected by increased lipid peroxidation biomarker 8-iso-PGF2α and also ROS-mediated protein oxidation and carbonylation [102]. Furthermore, our own research has reported that ingestion of energy-dense phytonutrient-reduced foods including cream, ice cream or sugar can increase plasma lipid peroxidation and reduce plasma total antioxidant capacity (TAC) 2–4 h after ingestion of these foods [107]. In another study, a 25% calorie restriction for 28 days in obese subjects was reported to reduce the lipid peroxidation biomarker F2- isoprostane [106]. Furthermore, long term calorie limitation studies on healthy human subjects have shown that fasting can reduce oxidative damage to DNA and decrease plasma concentrations of thiobarbituric acid-reactive species (TBARS), carbonylated proteins, otyrosine, and m-tyrosine, all indicators of oxidative damage to lipids, proteins and amino acids, respectively [104,105]. There are also reports on the effects of short-term calorie limitation (48 h) on reducing ROS generation by leucocytes [103]. Chronic calorie restriction can attenuate the age-associated increase in inflammatory IL-6 production by peripheral mononuclear cells [108] and also reduce plasma inflammatory CRP levels [109]. Several animal and human studies have also suggested the involvement of energy intake and calorie restriction (10–50% below usual ad libitum intakes) in delaying ageing and extending lifespan [110]. As a basis for these observations, it has been suggested that calorie restriction can affect longevity-linked transcripts along the insulin-like growth factor 1 (IGF-1)/insulin/FOXO pathway [111]. However, the exact mechanism responsible for these observations is yet to be fully understood. Furthermore, it is still not clear whether these effects are due to a simple reduction in energy intake or the associated reduction in specific dietary components such as protein [112,113].

#### 7.2.2. Protein Intake

Notwithstanding recommendations for an adequate intake of dietary proteins for the stimulation of muscle protein synthesis and/or suppression of protein breakdown [114], studies linking high intakes of proteins with ageing are emerging [115,116,117]. It has been suggested that protein restriction (40–85% of standard chow) can increase maximum lifespan by about 20% in rodents [115]. In humans, high protein intake (more than 20% of calories) has been associated with a 75% increase in overall mortality [117]. While suggested mechanisms include effects on ageing-associated molecules such as rapamycin (mTOR) [118], fibroblast growth factor 21 (FGF21), insulin [119], and insulin like growth factor 1 (IGF-1) [117], the mechanisms linking protein restriction to ageing are yet to be fully understood.

It has also been shown that protein restriction can independently reduce mitochondrial ROS generation, mitochondrial DNA oxidative damage biomarker 8-OHdG [120] and liver lipid peroxidation [121]. There are also reports that an increased protein intake can reduce the activity of the antioxidant enzyme superoxide dismutase in liver [121]. Protein intake and the metabolites of protein catabolism have also been shown to be negatively associated with the ageing-linked biomolecule, Nicotinamide adenine dinucleotide (NAD^+^), in a disease-free cohort [122]. Supplementation of the diet with specific amino acids (e.g., methionine) has also been shown to increase plasma lipid peroxidation biomarkers of hydroperoxides [123] and TBARS [124], and reduce tissue vitamin E levels [123].

#### 7.2.3. Processed Meat

Several studies suggest that the source of protein can be as important as the amount of protein in the diet in ageing, development of NCDs and promoting a state of oxidative stress [117,125,126]. In this context, it has been reported that high consumption of animal proteins specifically those derived from red meat was positively associated with plasma lipid peroxidation biomarker TBARs and was negatively associated with plasma antioxidant capacity [126]. Red meat is a complex medium containing high amounts of saturated fat, trans fat and heme-iron and its effect on promoting oxidative stress may be attributed, at least in part, to the cumulative pro-oxidant properties of these components [126,127]. Furthermore, cooking meat using high-temperature methods such as frying can produce highly pro-oxidant products such as advanced glycation end products (AGE), heterocyclic amines (HCAs) and polycyclic aromatic hydrocarbons (PAHs) [128,129,130,131]. Red meat is also a rich source of choline and l-carnitine, nutrients which can be metabolised to trimethylamine (TMA) by gut microbiota in colon, and then oxidised to trimethlyamine-*N*-oxide (TMAO) in the liver [132]. An elevated TMAO level as a result of high red meat intake has been shown to promote oxidative stress and vascular inflammation [133].

#### 7.2.4. Polyunsaturated Fatty Acids

Polyunsaturated fatty acids (PUFAs) are fatty acids that contain more than one double bond in their structure. According to the position of their first double bond from the methyl end of the carbon chain, PUFAs can be categorised into omega-3 FAs with the first double bond at the third carbon atom (e.g., alpha-linolenic acid (ALA), eicosapentaenoic acid (EPA) and docosahexaenoic acid (DHA)), and omega-6 FAs with the first double bond at the sixth carbon atom from the methyl end of the carbon chain (e.g., linoleic acid (LA) and arachidonic acid (AA)) [134].

Both omega-3 and omega-6 PUFAs are precursors to potent lipid mediator signalling molecules, termed “eicosanoids” with critical roles in the regulation of inflammation. Omega-3 PUFAs are precursors to anti-inflammatory eicosanoids such as the 3-series prostaglandins, thromboxane A3 (TXA3), and the 5-series leukotrienes (LTs), while omega-6 PUFAs are precursors to proinflammatory eicosanoids such as the 2-series prostaglandins, and thromboxanes (TXA2), and the 4-series of leukotrienes (LTB4) [135]. Depending on their abundance in the diet, these PUFAs often compete with each other for binding to the enzymes involved in eicosanoids formation including cyclooxygenase (COX) and lipoxygenase (LOX) [136]. Therefore, a diet rich in omega-6 PUFAs and low in omega-3 PUFAs can elevate omega-6 to omega-3 ratio with a pro-inflammatory eicosanoid profile promoting an inflammatory phenotype [137]. Further anti-inflammatory properties of omega-3 PUFAs include, negative regulation of the activity of enzymes involved in inflammatory eicosanoids production such as COX-1 [138], the activation of the G-protein receptor, and reducing the activation of NF-κB [138,139]. There are also reports of involvement of omega-3 PUFAs in attenuating oxidative stress by reducing oxidative stress-induced DNA damage in vascular endothelial cells, reducing intracellular ROS and increasing the mRNA levels of antioxidant molecules, such as heme oxygenase-1, thioredoxin reductase-1, and superoxide dismutase [140]. Supplementation with n-3 fatty acids has also been shown to reduce plasma F2-isoprostanes levels [141].

In spite of some evidence regarding the antioxidant properties of omega-3 PUFAs, it should be noted that both omega-3 and omega-6 PUFAs are chemically susceptible to oxidation and are major sites of oxidative attack in vivo due to their relatively large number of double bonds and the position of these bonds within their structure [142]. Oxidative modifications to omega-3 and omega-6 PUFAs result in the formation of lipid oxidation products (LOPs), including 4-hydroxyhexenal (4-HHE), and 4-hydroxynonenal (4-HNE), respectively [143]. Furthermore, oxidation of the omega-6 linoleic acids content of LDL-C during oxidation processes produces potent pro-oxidants such as 9-hydroxy-10,12-octadecadienoic acid (9-HODE) [144].

#### 7.2.5. Dietary Glycaemic Index

Dietary glycaemic index (GI) is a ranking of various carbohydrates according to the extent to which they raise plasma glucose levels after ingestion [145]. Consumption of foods with higher GI can contribute to either an acute or chronic elevation of plasma glucose, i.e., hyperglycaemia, which plays a significant role in developing oxidative stress and inflammation [146,147,148]. Hyperglycaemia induces oxidative stress and inflammatory processes via several well-known mechanisms, including high generation of superoxide anion by the mitochondria [149], glycation of antioxidant enzymes such as thioredoxin [150], activation of the polyol pathway, reducing plasma levels of NAD^+^ [151], activation of NF-κB [152], activation of protein kinase C [153], and increasing intracellular formation of the pro-oxidants such as advanced glycation end products (AGE) [154].

Several studies have shown the positive association between dietary GI assessed by a food frequency questionnaire during a 12-month period and plasma and urinary lipid peroxidation biomarkers of MDA and isoprostanes in healthy cohorts [147]. In healthy subjects after ingesting foods with different GI values, levels of the oxidative stress biomarker nitro-tyrosine and the activation of pro-inflammatory gene NF-κB were significantly higher in subjects in the high GI group compared to subjects in the low GI group [146]. Studies in diabetic subjects have also shown the effect of meals with high GI values on increasing plasma levels of MDA [151], CRP and TNF- α [155], and also raising LDL-C susceptibility to oxidation, as well as reducing total radical-trapping antioxidant parameter (TRAP) [151].

#### 7.2.6. Fruits and Vegetables

As rich sources of various antioxidants and vitamins (e.g., vitamin C and E), and a wide range of bioactive compounds known as phytochemicals (e.g., carotenoids), fruits and vegetables have long been recognised for their antioxidant and anti-inflammatory properties [156,157,158]. In this regard, several studies have shown the effects of high intakes of fruit and vegetables on reducing levels of oxidative stress and inflammatory biomarkers such as MDA [159], 8- isoprostane F2α [160], oxidised LDL-C [161], 8OHdG [162], CRP, interleukin receptor 1 (ILR1), IL6, and TNF-α [163]. In addition, consumption of a variety of fruits and vegetables has been reported to increase plasma and tissue levels of vitamin C and E, and also carotenoids such as lutein, zeaxanthin, α-carotene, β -carotene and lycopene, leading to a significant increase in plasma total antioxidant capacity (TAC) [164,165].

As lipid-soluble phytochemicals, carotenoids can also locate within biological membranes, and consequently, play a critical role in protecting cellular membranes as well as lipoproteins against damage by peroxyl free radicals [166,167]. It has also been shown that the intake of lycopene-rich vegetable products can prevent lipid peroxidation as indicated by decreased urinary F2-isoprostane and reduced LDL-C susceptibility to oxidation [168]. Furthermore, the intake of vitamins such as C and E from dietary sources including fruits, vegetables, nuts and seeds is negatively associated with plasma levels of oxidative stress biomarkers such as 8-isoprostane F2α and 8-oxo-7,8-dihydroguanine [169].

#### 7.2.7. Nutrient-Poor Foods

It is now widely promoted that a combination of dietary components can be more strongly associated with an inflammatory and oxidative stress profile than any single component considered individually [28]. Hence, the role of foods with complex matrices should also be taken into account in this context. Processed foods lacking nutrient density such as fast foods and sugar-sweetened soft drinks can still have a multifarious matrix rich in saturated and trans fatty acids, omega-6 PUFAs, sugar, and AGEs, which as mentioned previously, can all trigger the inflammatory response with consequent oxidative stress and cell damage [170]. In addition to increased oxidative damage regular consumption of these nutrient-poor foods can also lead to deficiencies in major micronutrients such as vitamins A, E, D, zinc and selenium which can weaken the body’s innate biochemical capability to neutralise the generated free radicals [171].

### 7.3. Common Social Drug Use

#### 7.3.1. Alcohol Intake

Alcohol is mainly metabolised into acetaldehyde by alcohol dehydrogenase in the liver [172]. However, it can also be metabolised by activation of the 2E1 isoform of the cytochrome P450 (CYP2E1) oxidase system which is induced during chronic alcohol consumption [173]. This process can lead to the generation of ROS and highly reactive free radicals including hydroxyl radicals and superoxide anions [174]. Chronic alcohol intake can cause hepatic accumulation of the hydroxyethyl radical and MDA, and deplete the cellular antioxidant glutathione (GSH) levels in rats [175]. Further studies in animals have revealed that high ethanol intake can increase liver 8-oxoguanine [176], and urinary 8-iso prostaglandin-F2α [177]. Studies in humans have shown that alcohol consumption in heavy alcohol drinkers can increase plasma levels of oxidised LDL-C [178], advanced glyoxidation end products (AGE) [179] and acetaldehyde-protein adducts, all of which are potent oxidants [180]. While high intakes of alcohol are consistently linked to wide spread tissue damage, a recent study by our group observed that chronic alcohol intakes equivalent to only 1–2 standard drinks a day were associated with a reduction in levels of total NAD(H), a recognised marker of intracellular metabolic and redox status, and an increase in levels of the inflammatory cytokine IL-6 in human cerebrospinal fluid (CSF) [181].

#### 7.3.2. Caffeine Intake

Caffeine (1,3,7-trimethylxanthine) is an alkaloid present in varying concentrations in coffee, tea, colas, energy drinks and chocolate [182,183]. While crude caffeine extract has been shown to possess limited lipophilic and hydrophilic antioxidant and cyclooxygenase-2 inhibitory activity, pure caffeine, appears to possess none of these attributes [184]. There is evidence that caffeine ingestion can increase lipid oxidation [185], enhance exercise-induced lipid peroxidation, indicated by plasma MDA and decrease plasma TAC levels in young men [186]. Furthermore, prenatal exposure to caffeinated energy drinks has been shown to induce oxidative stress and to increase lipid peroxidation in the liver, kidney and brain of newborn mice [187]. Caffeine, in either low or high concentrations has been reported to be significantly associated with a proinflammatory cytokine profile with elevated plasma levels of TNF-α, CRP [188], IL-1β [189], IL- 6, serum amyloid-A (SAA) [188], and soluble vascular cell adhesion molecule-1 (sVCAM-1) [190], and decreased serum levels of the anti-inflammatory cytokine IL-10 [189]. It has been suggested that caffeine produces these effects by inducing the expression of toll-like receptor 4 (TLR-4) and activating NF-κB pathways that increase the expression of inflammatory molecules [191,192].

#### 7.3.3. Cigarette Smoking

As a typical example of an oxidative stress-inducing compound, cigarette smoke contains more than 4000 different chemicals including oxidants and free radicals, many of which have established harmful effects [193]. Not surprisingly, cigarette smoking not only delivers oxidants and free radicals to the body, but also induces pro-inflammatory signalling via activation of NF-κB, which can exacerbate oxidative stress [194]. Not surprisingly therefore, smoking has been shown to be associated with high plasma levels of oxidative stress biomarkers such as oxidised LDL and isoprostane, and inflammatory biomarkers such as IL-1β, IL-6, IL-8, hs-CRP and soluble adhesion molecules [195].

### 7.4. Physical Activity

Emerging evidence suggests that regular physical activity, especially endurance training, can elevate antioxidant and anti-inflammatory biomarkers levels in the vasculature, the skeletal muscle and the circulation by various mechanisms [196,197]. Acute exercise causes a transient increase in ROS levels, providing the required stimulus for activating the antioxidant defence system. However, during chronic exercise this situation can be compensated for by upregulating antioxidant enzymes and thus, reducing the impact of free radicals production across the day [198]. In this context, several studies in animals and humans have shown that chronic exercise can increase serum total antioxidant status (TAS) [199], and antioxidant enzymes such as glutathione peroxidase [200] and haem oxygenase-1 (HO-1) [201] and reduce the levels of serum oxidative stress biomarkers, F2-isoprostanes [202], and MDA [196]. Regular exercise has also been shown to be associated with lower levels of several proinflammatory cytokines including interferon-γ (IFN-γ), IL-1β [203], CRP [204], IL-6 [202], IL-1β and TNF-α, and higher levels of anti-inflammatory molecules such as IL-4, IL-10, and transforming growth factor-β (TGF-β) [205].

### 7.5. Sleep and Tissue Oxygenation

It has been hypothesised that “good sleep” represents a state in which anti-oxidant potential is increased thereby improving the removal of free radicals generated during waking hours [206,207]. Experiments support the assertion that long-lasting wakefulness and sleep deprivation are associated with elevated oxidative damage and inflammation [208,209]. Increased levels of lipid peroxidation biomarkers such as myeloperoxidase-modified LDL-C [208], reduced levels of antioxidant enzymes such as glutathione peroxidase, catalase, and superoxide dismutase [210], and elevated production of pro-inflammatory molecules including IL-1β, IL-6, IL-17 [211] and high sensitivity C-reactive protein (hs-CRP) [212] have been reported in several animal and human studies.

One of the most common sleep-related disorders that represent a key public health problem is obstructive sleep apnoea (OSA). OSA is defined by repetitive episodes of upper airway collapse during sleep resulting in nocturnal intermittent hypoxia with the consequent induction of oxidative stress [213,214]. In this context, experiments in patients with OSA have revealed excessive superoxide radical production from leukocytes [215] and reduced bioavailability of nitric oxide (NO) the main endothelial-derived vasodilator linked to oxidative stress [216]. Furthermore, increased serum concentrations of TBARs [217] and exhaled breath 8-isoprostane [216], increased urinary 8-OHdG [218], and higher plasma advanced oxidation protein products [219] have been reported. There is also evidence for decreased plasma total antioxidant status (TAS), vitamin A and E [220], and reduced plasma levels of antioxidant enzyme, superoxide dismutase [221], and paraoxonase-1 [217] in OSA. Several studies have also suggested that the intermittent hypoxia in OSA can stimulate inflammatory pathways [222]. In this context, OSA patients have shown increased plasma levels of TNF-α, and IL-8 consistent with the extent of hypoxia during sleep [223].

### 7.6. Psychological Factors (Stress, Anxiety and Depression)

Mild anxiety is a normal response to psychological stress. However, when such responses become extreme and persistent, it is considered pathologic and is referred to as anxiety disorder (AnxD) [224]. AnxDs are implicated in a number of psychiatric disorders, in particular depression [225]. It is well known that oxidative stress and inflammation play a major role in both initiation and progression of these diseases [226,227]. Elevated plasma levels of ROS [228,229] and oxidative stress biomarkers such as MDA [230], 8- iso-prostaglandin F2 [231], and 8-OHdG [232] are important features of AnxD and major depression. Furthermore, studies have also reported decreased plasma TAC [233], reduced activity of antioxidant enzymes such as glyoxalase 1 (GLO1) [234] and reduced plasma levels of antioxidants such as vitamin E [235], GSH [236] and coenzyme Q10 [237] in these disorders.

Anxiety and depression are also accompanied by an increased inflammatory profile as indicated by elevated serum levels of a variety of pro-inflammatory cytokines such as IL-6 [231], TNF-α [233] and CRP [238]. There is also evidence for the induction of inflammatory enzymes such as indoleamine 2,3-dioxygenase (IDO), a rate-limiting enzyme for the metabolism of tryptophan via the kynurenine pathway [239,240]. Increased catabolism of tryptophan by IDO can lead to depletion of antioxidants like tryptophan and its metabolite serotonin (5-hydroxy tryptophan), and also increased synthesis of the pro-oxidant tryptophan catabolites (TRYCATs), quinolinic acid and kynurenine [241].

**Figure 3:**
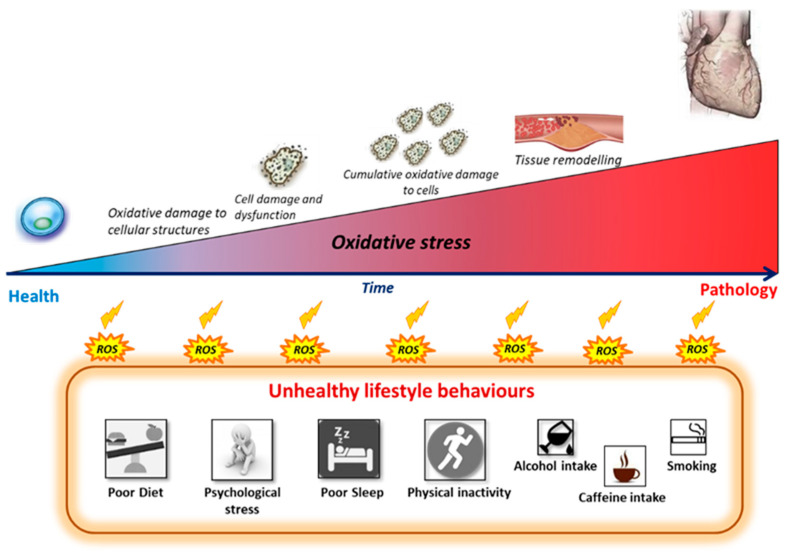
Progressive oxidative damage processes induced by chronic exposure to unhealthy lifestyle behaviours. Adherence to unhealthy lifestyle behaviours can gradually shift the body’s redox balance toward a state of oxidative stress with chronic subclinical damage to cells, tissues and organs. Oxidative damage plays a major role in the onset and development of non-communicable disease such as cardiovascular disease. ROS: Reactive oxygen species.

A model that develops from these observations suggests that unhealthy lifestyle behaviours trigger increased oxidative activity (via several sources, including inflammation) leading to an often-chronic state of redox imbalance (oxidative stress) that gradually shifts the body’s biochemistry toward a diseased phenotype. If unhealthy lifestyle behaviours persist, the resulting biochemical adaptation will, over a period of time, drive the body to a state of disease [242,243,244] (Figure 3).

## 8. Evidence that Changes in Lifestyle-Linked Oxidative Stress Is Detectable in Disease-Free Humans

As mentioned, several studies have shown significant associations between either individual lifestyle behaviours or composite lifestyle scores, and oxidative stress and inflammatory biomarkers and risk of various NCDs such as CVDs [31,32,242,245,246,247,248]. However, though oxidative stress and inflammation have been demonstrated clearly in the presence of disease, there are currently limited data showing a relationship between lifestyle behaviours, subclinical changes in oxidative stress and inflammatory biomarkers and changes in classic disease markers during the stage where no clinical symptoms of disease are present. In other words, few studies have investigated whether changes in oxidative stress and inflammatory biomarkers are observable in otherwise healthy persons in which “unfavourable” lifestyle behaviours are nonetheless present. Even though some authors have suggested the measurement of oxidative stress and inflammatory biomarkers be included in routine analysis performed in clinical laboratories [249,250,251,252,253,254], they were generally focused on disease management, and methodological aspects, rather than disease prevention. In addition, they provided no link to lifestyle behaviours as the main underlying cause of disease. Hence, additional work is required to further develop the concept and identify the potential benefits of monitoring levels of oxidative stress and inflammatory biomarkers both in terms of its value to early identification of NCD pathologies and its link to multiple lifestyle behaviours in apparently healthy individuals.

Among the few studies available in this context, results from our research group have shown that lifestyle-associated changes in oxidative stress and/or inflammatory biomarkers can indeed be detectable even in humans with no clinical symptoms of disease [255]. Thus, indicating that changes in oxidative inflammatory activity may be present during the prodromal or developmental phase of the disease process. In this study, we showed that a simple measure of redox balance, the ratio of total antioxidant capacity to hydroperoxide ratio (TAC/HPX ratio), was sensitive enough to be significantly associated with lifestyle behaviours including body fat percentage, sleep quality, sleep apnoea, depression and dietary intakes of red meat and caffeine, in healthy humans [255]. Lipid hydroperoxide (HPX) has been shown to be a reasonably stable, ubiquitous and sensitive by-product of lipid peroxidation [249,256,257]. Due to their propensity to contain double bonds, membrane-associated lipids appear to be very vulnerable to free radical insults, and thus biomarkers associated with lipid peroxidation have been shown to be more sensitive than other markers such as protein oxidation products [258]. The coupling of HPX with a measure of total antioxidant capacity (TAC), an integrated measure of all antioxidants present in plasma [259], provides an index of the degree of ongoing free radical damage, relative to available antioxidant resources, as it relates to the plasma. Thus, the TAC/HPX ratio provided an added degree of sensitivity in detecting an otherwise healthy individual’s specific changes in redox balance in response to their set of lifestyle challenges [255].

Importantly, these findings also showed that the plasma redox potential could have clinical relevance as the TAC/HPX ratio was independently associated with NCD risk and changes in classic CVD risk markers such as LDL-C, total cholesterol and fasting plasma glucose (FPG) [255]. Similar relationships had been previously reported by Brunelli et al. (2017) who showed that plasma antioxidant capacity was significantly associated with traditional cardiovascular risk factors, including total cholesterol and total cholesterol to HDL-C ratio, in a healthy population [260].

Using an otherwise healthy cohort, we also observed that the TAC/HPX ratio was associated with the Framingham risk score (FRS), an established CVD risk predictor, which was independent of the conventional CVD risk markers such as LDL-C, HDL-C and blood pressure [255]. This association is consistent with earlier reports in which FRS was associated with other serum oxidative stress-linked biomarkers such as peroxiredoxin [261], γ-glutamyltransferase (GGT) [262], bilirubin [263], glutathione redox state [56], oxidised disulphide cysteine [264], oxidised phospholipids [265], oxidised LDL [266], 9-hydroxy eicosatetraenoic acids, and F2-isoprostanes [57]. Collectively, these data suggested that changes in plasma redox balance, especially in the preclinical stage when the person is assumed to be healthy, may reflect an individual’s early biochemical shift toward disease. However, information from future longitudinal studies that include a comprehensive range of inflammatory and oxidative markers will be needed to verify this hypothesis.

As previously outlined, lifestyle behaviours are recognised as major factors influencing the subclinical risk indicators of NCDs such as CVD changes in carotid intima-media thickness (CIMT) in the general population [267,268,269]. The critical role of oxidative stress and inflammatory processes in the development of vascular abnormalities and increased CIMT has been established in several studies in clinical cohorts [18,270]. Interestingly, the results from the same healthy cohort in our lab also showed that the collage of lifestyle behaviours could link to increased risk of CIMT independent of conventional CVD risk markers. In other words, changes in an integrated measure of lifestyle behaviours were shown to be strongly associated with changes in CIMT and at the same time with plasma biomarkers of redox balance and oxidative stress [271]. As this measure of lifestyle behaviours was also associated with oxidative stress processes in our cohort with no clinical signs of disease, it can be inferred that oxidative stress may play an independent role in the initiation of subclinical abnormalities involved in the development of CVD. Therefore, relying on conventional biomarkers such as LDL-C to estimate the risk of disease without considering the redox state might not be sensitive enough to identify the actual risk of developing disease for individuals whose redox state is responding poorly to their constellation of lifestyle behaviours. These findings are consistent with results of other studies that reported independent associations between oxidative stress and CIMT in disease-free individuals [18,56].

## 9. Clinical Application of Monitoring Oxidative Stress and Inflammation

**Figure 4:**
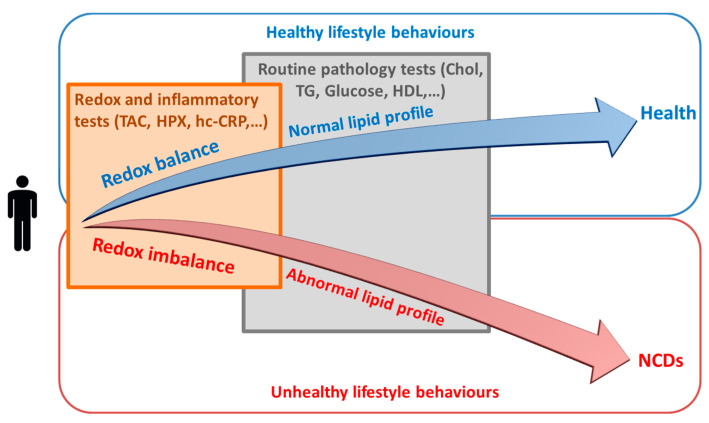
Assessment of redox state (i.e., assessment of the degree to which oxidative stress is present) as a possible approach for early distinction of whether various lifestyle behaviours are driving an individual toward health or toward development of disease-related pathologies. NCDs: non-communicable diseases; TAC: total antioxidant capacity; HPX: lipid hydroperoxides; hc-CRP: high sensitivity C-reactive protein; Chol: cholesterol; TG: triglycerides; HDL: high density lipoprotein.

Collectively, current available evidence suggests that, oxidative stress and inflammatory pathways represents a sensitive shared molecular pathway between various lifestyle behaviours through which these behaviours may gradually drive an individual’s biochemistry/physiology toward a state of developing disease. Consistent with this hypothesis there is a developing body of evidence showing that oxidative stress-associated changes are detectable in apparently healthy individuals in association with specific lifestyle behaviours [255,271]. Thus, the measurement of biomarkers associated with oxidative stress (and inflammation), may help with predicting early shifts toward the development of NCDs and hence may enhance the ability to correctly characterise an individual’s disease trajectory. Therefore, monitoring oxidative stress and inflammatory biomarkers and/or including a measure of oxidative stress and inflammation in any risk assessment tool may provide an early and more direct assessment of the presence of damage processes that go beyond the conventional downstream risk factors currently used for predicting disease risk. An appropriate clinical application of this concept is that it may provide an early indication of whether an individual’s biochemistry is driving him/her toward health or disease (Figure 4). On-time distinction of this trajectory can enable the initiation of appropriate modifications (mainly lifestyle-related) and assist with a better health outcome in terms of prevention of chronic NCDs.

This may have some enhanced discriminating capacity for those at risk of disease as it will identify individuals whose biochemistry is not responding well to their collage of lifestyle choices and therefore are in the process of developing an NCD. Assessing NCD risk using (or at least including) oxidative stress and inflammatory markers is therefore increasing the focus on upstream causative metabolic processes rather than the less specific downstream risk factors such as body weight and lipid levels.

It is relevant to reiterate that a condition of oxidative stress and inflammation is not a diseased state but is rather a preclinical state representing significant cellular stress and damage [18,56,264,272]. The information and perspective presented in this paper supports the view that this state can be produced as a result of chronic exposure to various unhealthy lifestyle behaviours. The damage caused by oxidative stress and inflammation, if not detected early and prevented, can accumulate over time causing serious tissue remodelling that will eventually result in a diseased phenotype [15,16,242]. Therefore, the assessment of redox state during the early stage when no clinical symptoms of disease are manifest can assist with identifying an individual’s early biochemical shifts toward development of disease. In this way, the ongoing tissue damage due to chronic oxidative stress and inflammation can be reduced or even halted by early modification strategies such as lifestyle changes. This will prevent or limit development of irreversible organ damage and subsequent disease. Improving the course of preclinical disease in this way not only can provide improved patient outcomes but also can reverse disease progression and, therefore, reduce both the need and costs of ongoing healthcare.

**Figure 5:**
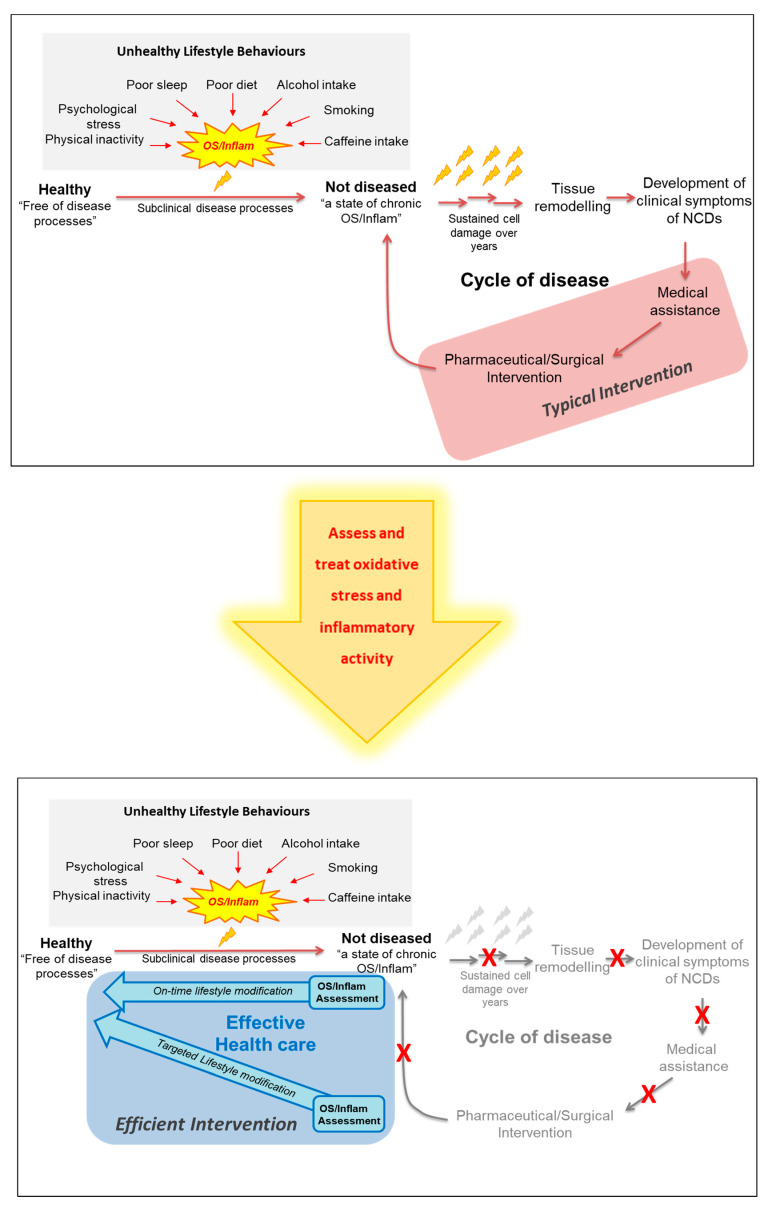
A suggested model for a more effective approach to treatment/prevention of the non-communicable diseases. NCDs: non-communicable disease; OS: oxidative stress; Inflam: Inflammation.

When fully developed, this approach may offer a way to bridge the gap between the preventive aims of population-based public health strategies and the therapeutic goals of patient-centred clinical practices to more favourably address individuals wellness needs (Figure 5).

## 10. Monitoring Oxidative Stress and Inflammation

As mentioned, our research group previously reported that different lifestyle behaviours are associated with changes to the oxidative stress biomarkers TAC/HPX which were detectable in a human cohort with no clinical symptoms of disease [255]. In addition we showed that changes in redox balance (based on lifestyle) correlated with the TAC/HPX biomarkers and with changes in the CVD risk factor CIMT [271]. These observations are consistent with the hypothesis that OS/inflammatory markers are early indicators of developing NCDs and if monitored, can enable effective treatment strategies in the preclinical disease phase before irreparable tissue damage occurs.

Unfortunately, a clinically validated and universally accepted profile of oxidative stress (and to some extent inflammatory) biomarkers has not yet been established in the clinical context. However, a wide variety of biomarkers and analytical methods have been developed to measure oxidative stress and inflammation in the research and clinical arenas. While there are popular clinical markers of inflammation (e.g., hsCRP) clinically validated markers of oxidative stress are still not apparent. As a general recommendation, any analytical method for measuring OS and inflammation should be accurate, sensitive, specific, reproducible, and interference-free [273]. Furthermore, to promote their use in clinical settings, the methods should be based on a simple and quick protocol, and involve low cost. Considering the highly reactive nature of oxidative species and their short half-life, this is a formidable challenge and care needs to be taken to ensure the stability of the sample to avoid artefactual oxidation. Suitable analytes must be able to be stabilised in the clinical environment, present in quantifiable concentrations and measurable within the detection range of a reliable analytical method across populations, allowing the establishment of reference values. The analyte should also ideally be a specific and major product of oxidative/nitrosative damage [273,274]. To our knowledge, no individual oxidative stress biomarker meets all of these technical requirements. However, some are better than others and a select panel of currently available markers will likely provide the most clinically useful information [274]. While it is recognised that further development in this area is needed, we have listed a selected range of biomarkers used for detecting oxidative stress and inflammation that are adaptable to the clinical environment (Table 1 and Table 2) [252,275,276,277,278,279,280,281,282,283,284,285,286,287,288,289]. With careful selection of markers and consideration of the points mentioned above, erroneous interpretations can be minimised and an accurate profile of an individual’s biochemical redox health status should be obtainable and able to be applied to the (pre)clinical setting.

## 11. Conclusions

Current literature supports the view that lifestyle-induced oxidative stress and inflammation are primary drivers of metabolic imbalances that cause NCDs. Oxidative stress and inflammation produce a preclinical state of progressive tissue damage that, if continued, will result in selected system failures and accompanying disease. Effective management or prevention of the chronic subclinical tissue damage may be possible if levels of the relevant oxidative stress and inflammatory biomarkers are quantified and monitored and their link to an individual’s unique set of lifestyle behaviours are identified. The construction of such a wellness assessment profile would serve as an early alarm for the apparently healthy individual who is on a path to disease. This assessment of oxidative and inflammatory markers could also provide a baseline from which the success of any intervention can be monitored. Such a modified clinical approach has the potential to improve the effectiveness of current public health strategies to both prevent and reverse NCDs and their accompanying economic burden.

## Figures and Tables

**Table 1:** Potential biomarkers of oxidative stress (OS).

OS Marker	Advantages	Disadvantages	Reference
IsoPros	Can be detected in various samples (serum, plasma urine) and has been shown to be elevated in the presence of a range of cardiovascular risk factors and cancer	Evidence in clinical disease still developing. Test available in specialist labs only	[275]
MDA	Easy to quantify in serum, plasma or urine. Studies show MDA can predict progression of coronary artery disease and carotid atherosclerosis at 3 years.	TBARS assay is non-specific (can detect aldehydes other than MDA) therefore potential for interferences. Test available in specialist labs only.	[252]
Hydroperoxides (HPX)	Lipid hydroperoxides are non-radical intermediates of lipid peroxidation. If frozen on collection, relatively stable in serum/plasma and can be transported. Growing body of evidence for correlation with clinical disease. Commercial test kits available	Can undergo reductive degradation if not stored properly which will decrease levels.	[276]
Nitrotyrosine	Human studies have demonstrated association with coronary artery disease independent of traditional risk factors	Circulating levels not equivalent to tissue levels. Current detection methods are expensive and impractical for scaling up to the clinical setting.	[277]
MPO	Measured in serum or plasma. Commercial assays available. Strong evidence that MPO correlates with cardiovascular disease risk	Influenced by sample storage and time to analysis. Test available in specialist labs only.	[278]
oxLDL	Serum or plasma levels routinely available as a clinical marker in many larger routine pathology labs. Elevated in coronary artery disease, increasing oxLDL correlates with increasing clinical severity. Also is predictive of future coronary artery disease in healthy population. Good reproducibility from frozen samples	Ox LDL has been studied mostly in relation to CVD so results not generalisable to other NCDs at this stage	[279]
Total antioxidant capacity (TAC)	Plasma levels considered a good measure of the activity of the low molecular weight, antioxidants, including albumin, Vitamin C, SH group containing antioxidants (glutathione), polyphenolic and carotenoid compounds and bilirubin. Has been correlated to a variety of disease states. Commercial testing kits available	The specific contribution of antioxidant enzymes such as catalase and SOD are excluded	[280]
Gene expression	The change in expression of various genes may reflect altered redox balance and can be measured simultaneously using microarray technology	Expensive and It is unclear if expression profiles of cells in biological samples reflect that in clinical tissues. Test only available form specialist labs	[281]

Abbreviations: IsoPros (F2 Isoprostanes), MDA (malondialdehyde), HPX (hydroperoxide), MPO (myeloperoxiodase), oxLDL (oxidised low density lipoprotein).

**Table 2:** Potential biomarkers of inflammation.

Inflammation Linked Marker	Description/Advantage	Disadvantages	Reference
IL-6	Early release pro-inflammatory cytokine produced in response to infections and tissue injuries	Half life of 10 min. Therefore, only elevated during active inflammatory activity. Can also have anti-inflammatory effects. Test only available in specialist labs	[282]
TNF-α	Pro-inflammatory cytokine produced by macrophages and lymphocytes	Half life of 18 min. Therefore, only elevated during active inflammatory activity. Test only available in specialist labs	[282]
IL-1β	Pro-inflammatory cytokine and lymphocyte/monocyte activating factor	Half life of <4 h. Therefore, only elevated during active inflammatory activity. Test only available in specialist labs	[282]
IL-8	Pro-inflammatory cytokine produced by polymorphonuclear (PMN) cells	Half life of ~4 h. Therefore, only elevated during active inflammation due largely to bacterial infections. Test only available in specialist labs	[282]
IL-10, IL-4	Anti-inflammatory cytokines. IL-4 produced by activated T cells, mast cells, basophils, eosinophils, and Natural killer T cells. IL-10 produced by macrophages, dendritic cells (DC),3 B cells, and various subsets of CD4+ and CD8+ T cells	Half life of <4.5 h. Therefore, only elevated during active inflammatory activity. Test only available in specialist labs	[282]
Fibrinogen	Pro-inflammatory regulation. High levels well documented risk for many inflammatory conditions. Test routinely available in pathology labs	The mechanistic links between coagulation and inflammation involving fibrinogen activation and signalling are not yet fully known	[283]
Alb/Glob ratio	Decreased in inflammation. Sensitive indicator of inflammatory disease, in particular kidney disease. Test available in routine pathology labs	Ratio may change for reasons other than inflammation, e.g., nutritional deficiencies such as low protein intakes	[284,285]
Tryp/Kyn ratio	Sensitive indicator of T-helper 1 (i.e., IFN-γ) immune responses. Reduced ratio reported in a range of inflammatory associated conditions including aging, cancer, rheumatoid arthritis and CVD. Altered Kyn:Tryp ratio remains longer and fluctuates less than individual cytokines.	Test not routinely available outside of research labs	[286]
Neopterin	Macrophage activation product dependent on IFN-γ activation. Levels remain elevated for longer after IFN-γ levels return to normal.	Produced secondary to IFN-γ activation of macrophages. Test only available in specialist labs.	[287]
hs-CRP	Acute phase protein produced by liver following IL-6 activation. Widely used as a clinical marker of inflammation. Levels remain elevated longer than IL-6 and other cytokines. Test widely available in routine pathology labs.	As an acute phase protein levels rise by 2 h peak by 48 h. Half life 18 h. However, one of the 2 isoforms of CRP may have anti-inflammatory effects	[288]
Omega-3 Index/ratio	Primary ant-inflammatory modulators. Large body of evidence linking reduced omega-3 levels to conditions associated with chronic inflammation including rheumatoid arthritis, CAD and depression.	Indirect marker of inflammation potential. Test only available in specialist labs	[289]

Abbreviations: IL-6 (interleukin-6), TNF-α (tumour necrosis factor alpha), IL-1β (interleukin 1-bets), IL-8 (interleukin 8), IL-10, IL-4 (interleukin 10 and interleukin 4), Alb/Glob ratio (albumin:globulin ratio), Tryp/Kyn ratio (tryptophan:kynurnein ratio), hs-CRP (high sensitivity C-Reactive protein), CVD (cardiovascular disease).
